# The more, the healthier: Tree diversity reduces forest pests and pathogens

**DOI:** 10.1371/journal.pbio.3002525

**Published:** 2024-02-28

**Authors:** Felicia Keesing, Richard S. Ostfeld

**Affiliations:** 1 Program in Biology, Bard College, Annandale-on-Hudson, New York, United States of America; 2 Cary Institute of Ecosystem Studies, Millbrook, New York, United States of America

## Abstract

How frequently, and under what conditions, does biodiversity reduce the transmission of infectious diseases through “dilution effects”? This Primer explores a new PLOS Biology study of forest pests that provides strong evidence for their generality.

## Teaser

How frequently, and under what conditions, biodiversity reduces disease through “dilution effects” has been a subject of ongoing research. A new study of forest pests in *PLOS Biology* provides strong evidence for their generality.

In ecosystems around the world, the loss of biodiversity has been found to increase the risk of infectious diseases. This phenomenon occurs because the taxa most likely to transmit parasites often thrive when natural biodiversity is lost. Because biodiversity prevents this proliferation, these phenomena are called dilution effects, and they have been documented for diseases of humans, wildlife, and both domesticated and wild plants [1].

Two key questions about dilution effects have been how frequently, and under what conditions, they are likely to occur. To answer these questions, a number of researchers have now conducted meta-analyses of individual studies that examine the relationship between biodiversity and infectious diseases. These meta-analyses consistently show that dilution effects are observed much more frequently than expected by chance and that they are equally common and equally strong across a variety of disease systems. One criticism of these meta-analytic approaches, however, is that the quality of their conclusions depends on the quality of the pool of studies on which they draw. For example, if researchers tend to study specific disease systems that are likely to show dilution effects, then a meta-analysis will conclude a greater tendency for dilution effects than would be found if disease systems were chosen at random. Conversely, some meta-analyses have been criticized for establishing criteria that exclude studies that demonstrate dilution effects. These so-called “cherry-picking” problems have generated heated debate in disease ecology.

An alternative approach to determining how frequently dilution effects occur is to assess the frequency of dilution effects in a single ecosystem, considering the effects of biodiversity on a suite of parasites that co-occur in a particular habitat. This type of approach is less common, but extremely informative. In a large-scale grassland experiment in Germany, for instance, Rottstock and colleagues [2] found that plant diversity reduced the prevalence of all but one type of plant pathogen, or microparasite.

**Fig 1:**
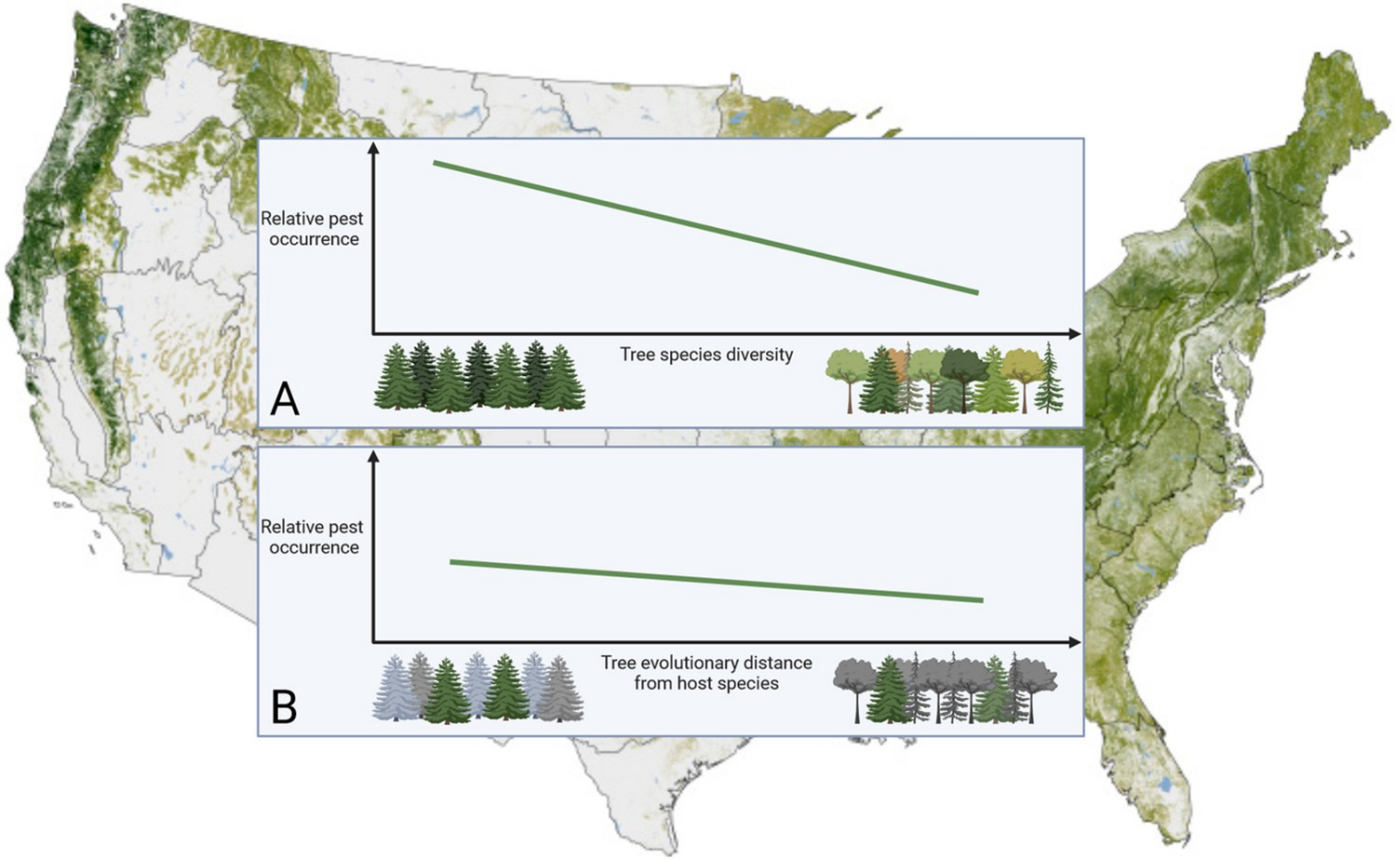
Conceptual model depicting how relative pest occurrence on >25,000 forested plots across the United States (**A**) declines with forest tree diversity and (**B**) by the degree of relatedness of tree communities to the host tree species (shown in green in **B**), all else being equal. Image created in BioRender. NASA Earth Observatory map by Robert Simmon, with data sets compiled and analyzed by the Woods Hole Research Center, using data from the Shuttle Radar Topography Mission, the National Land Cover Database, and the Forest Inventory and Analysis of the US Forest Service.

In this issue, Gougherty and Davies [3] present a third, and compelling, approach to assessing the frequency of dilution effects. Using data from forest surveys conducted by the United States Department of Agriculture (USDA) Forest Service, Gougherty and Davies compiled a record of the prevalence of damage inflicted by 60 forest pests and pathogens in more than 25,000 forest plots across the US. They found that more diverse forests had lower prevalence of damaged trees, demonstrating an overarching dilution effect (Fig 1A). Dilution effects were not uniform for all types of pests, though. For some, like butternut canker, forest tree diversity strongly reduced the prevalence of damage, while for others, like the spongy month *Lymantria dispar*, tree diversity had no effect.

Using Bayesian hierarchical models, Gougherty and Davies asked whether features of either the forests or the pests predicted when dilution effects occurred. Whether pests were native or introduced did not affect pest prevalence, nor did the degree to which pests were specialized on particular host species. However, the phylogenetic relatedness of the trees in the forest plots did modify the strength of dilution effects. In plots where the average tree in a community was more distantly related to the known host trees for a given pest, dilution effects were stronger (Fig 1B). This observation suggests that distantly related trees might be less susceptible to spillover of a particular pest and thus more likely to reduce transmission between individuals of the host species. A similar effect of phylogenetic relatedness has been observed for infectious diseases with animal hosts, including highly pathogenic avian influenza in Europe [4].

The study by Gougherty and Davies focuses on tree pests, a broad grouping that includes traditional macroparasites (for instance, the southern pine beetle, *Dendroctonus frontalis*) and microparasites (also called pathogens, including the fungus *Sirococcus clavigignenti-juglandacearum*, which causes butternut canker). The grouping “forest pests” includes a third type of organism as well—arthropods that consume tree tissues but are generally classified as herbivores rather than parasites. Spongy moths (*Lymantria dispar*) are an example of this type of pest; their effect on trees occurs primarily as a result of defoliation by their caterpillar stage. Prior studies of dilution effects have focused on how diversity impacts macro- and microparasites, but there is evidence that diversity can reduce herbivorous pests as well [5].

Gougherty and Davies’ rigorous assessment of the massive USDA data set demonstrates that dilution effects are pervasive for tree pests in forests of the US. Taking the results from these various approaches together, we have an increasingly strong body of evidence demonstrating that dilution effects are widespread and common. This synthesis allows disease ecologists to focus on other important questions, including the consequences of reduced parasite transmission in diverse ecosystems. For example, Wang and colleagues [6] recently demonstrated that dilution effects in communities of soil microbial pathogens help explain patterns of plant productivity.

As Gougherty and Davies [3] point out, we should expect greater pest problems in forests with low diversity, whether that low diversity results from natural or human causes. Their findings suggest several avenues for future research. For instance, focusing on the protection of phylogenetic diversity within communities could promote stronger suppression of pests than simply focusing on the protection of species diversity alone. If specific conservation strategies are targeted toward maintaining a specified level of species richness (the number of species), without attention to the phylogenetic diversity (the degree of evolutionary relatedness) represented in that community, these might be less effective at reducing pest transmission as well.

In an ironic twist, the new work of Gougherty and Davies [3] relies on the USDA’s Forest Inventory and Analysis (FIA) database. The FIA was funded through the McSweeney-McNary act of 1928, which directed the USDA to inventory the nation’s forests to facilitate extraction of forest resources, especially timber. Almost 100 years later, this rich database has become a resource for scientists and land managers trying to protect those same forests now and into the future.
